# What visual illusions tell us about underlying neural mechanisms and observer strategies for tackling the inverse problem of achromatic perception

**DOI:** 10.3389/fnhum.2015.00205

**Published:** 2015-04-21

**Authors:** Barbara Blakeslee, Mark E. McCourt

**Affiliations:** Department of Psychology, Center for Visual and Cognitive Neuroscience, North Dakota State UniversityFargo, ND, USA

**Keywords:** brightness, lightness, contrast, inferred-lightness, achromatic perception

## Abstract

Research in lightness perception centers on understanding the prior assumptions and processing strategies the visual system uses to parse the retinal intensity distribution (the proximal stimulus) into the surface reflectance and illumination components of the scene (the distal stimulus—ground truth). It is agreed that the visual system must compare different regions of the visual image to solve this inverse problem; however, the nature of the comparisons and the mechanisms underlying them are topics of intense debate. Perceptual illusions are of value because they reveal important information about these visual processing mechanisms. We propose a framework for lightness research that resolves confusions and paradoxes in the literature, and provides insight into the mechanisms the visual system employs to tackle the inverse problem. The main idea is that much of the debate and confusion in the literature stems from the fact that lightness, defined as apparent reflectance, is underspecified and refers to three different types of judgments that are not comparable. Under stimulus conditions containing a visible illumination component, such as a shadow boundary, observers can distinguish and match three independent dimensions of achromatic experience: apparent intensity (brightness), apparent local intensity ratio (brightness-contrast), and apparent reflectance (lightness). In the absence of a visible illumination boundary, however, achromatic vision reduces to two dimensions and, depending on stimulus conditions and observer instructions, judgments of lightness are identical to judgments of brightness or brightness-contrast. Furthermore, because lightness judgments are based on different information under different conditions, they can differ greatly in their degree of difficulty and in their accuracy. This may, in part, explain the large variability in lightness constancy across studies.

## Introduction

A central question in the study of human visual perception is how and under what circumstances the visual system is able to separate the physically invariant reflectance of a surface R(x, y) from its potentially changing illumination I(x, y). The visual system does not have direct access to either reflectance (R) or illumination (I) but only to their product which determines the luminance (intensity) distribution falling on the photoreceptor array: L(x, y) = I(x, y) • R(x, y). The independent recovery of surface reflectance R(x, y) and illumination I(x, y) is thus an ill-posed (inverse) problem in that there are innumerable combinations of these two variables that can give rise to any particular intensity distribution, and in the absence of additional information it is impossible to uniquely recover the physically correct solution. Current research centers on understanding the prior assumptions and processing strategies the visual system uses to parse (correctly or incorrectly) the retinal intensity distribution (the proximal stimulus) into the surface reflectance and illumination components of the scene (the distal stimulus—ground truth). It is generally agreed that the visual system must compare different regions of the visual image in its attempt to solve or approximately solve this problem; however, the nature of these comparisons and the mechanisms underlying them are topics of intense debate. Perceptual illusions are of value in this regard because they reveal important information about these underlying visual processing mechanisms.

**Figure 1:**
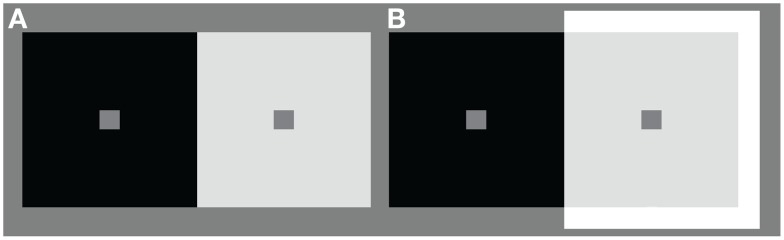
**Simultaneous contrast stimulus. (A)** Illustration of a reflectance edge and **(B)** an illumination edge simultaneous brightness/lightness contrast stimulus. In **(A, B)** all of the test patches have the same luminance. The low luminance and high luminance backgrounds are also equivalent in the two stimuli. Stimulus **(A, B)** differ only in the addition of a far surround to three sides of the high luminance background in **(B)** to simulate an illumination edge.

The debate concerning the mechanisms underlying the perceptual correlates of the physical components of the visual stimulus, i.e., apparent intensity (brightness), apparent reflectance (lightness), and apparent illumination, is historically longstanding and well-illustrated by the illusion variously referred to as simultaneous brightness (or lightness) contrast (Figure 1). Note that the different names for this illusion (as well as the different adjectives used to describe it) reflect the historical controversy concerning its cause, i.e., whether the illusion is one of apparent intensity (brightness) or apparent reflectance (lightness). In an attempt to avoid confusion, or the erroneous impression that these names refer to different illusions, we use the term simultaneous contrast and describe the illusion using both the descriptors used to refer to the dimensions of apparent intensity (brightness, ranging from dim to bright), and apparent reflectance (lightness, ranging from black to white). Accordingly, simultaneous contrast can be described as an illusion in which a mid-intensity (gray) test patch on a dim (black) background looks brighter (whiter) than an identical test patch on a bright (white) background (Figure 1A).

Nearly 150 years ago Mach (1865, in Ratliff, 1965) and Hering (1874/1964) advocated what has come to be called a “low-level” account of simultaneous contrast based on reciprocal interactions between elements in the nervous system. This view was later represented by early models of lateral inhibitory interactions such as those performed by the center-surround receptive fields of neurons in the retina and lateral geniculate nucleus (LGN) of cats and primates (for reviews see: Heinemann, 1972; Jameson and Hurvich, 1989; Kingdom and Moulden, 1989; Fiorentini et al., 1990; Kingdom, 1997, 2003, 2011, 2013; Fiorentini, 2003) and is today represented by modern multiscale filtering models such as the ODOG model (Blakeslee and McCourt, 1999; Robinson et al., 2007), as well as by models positing the filling-in (Grossberg and Todorovic, 1988; Rossi and Paradiso, 2003) of the representation of a test patch by information extracted by relatively high-frequency filters (small receptive fields) at the edges of the patch or at multiple edges within the stimulus (Rudd, 2010, 2013, 2014).

von Helmholtz (1866/1962), on the other hand, emphasized the importance of “unconscious inference” in producing the simultaneous contrast effect. According to Helmholtz observers interpret the test patch on the bright (white) background to be under a higher level of illumination than the equiluminant test patch on the dim (black) background. Since the mid-intensity (gray) test patches reflect the same amount of light to the eye, Helmholtz proposed that the visual system “unconsciously infers” that the more highly illuminated test patch is less reflective resulting in its appearance as dimmer (or a darker shade of gray). This relatively “high-level” account is often revisited in current explanations in which a variety of “mid-level” factors such as junctions (Adelson, 2000) or Gestalt grouping (Gilchrist et al., 1999; Gilchrist, 2006), lead to perceptual inferences about depth, illumination, reflectance, and transparency (Gilchrist et al., 1999; Kingdom, 1999, 2011, 2013; Logvinenko, 1999; Adelson, 2000; Logvinenko and Ross, 2005; Bressan, 2006; Gilchrist, 2006; Anderson and Winawer, 2008; Radonjic et al., 2010; Radonjic and Gilchrist, 2013). Helmholtz's ideas are also echoed by proponents of the “empirical” approach that replaces “unconscious inference” with the idea that percepts arise in proportion to their respective frequencies of occurrence in the past experiences of both the species and the individual observer (Purves et al., 2004).

## Proposal of a common theoretical framework for lightness and brightness perception

In a recent review of the brightness/lightness literature Kingdom (2011) noted that “Divided into different camps, each with its own preferred stimuli, methodology and theory, the study of LBT (lightness, brightness, transparency) is sometimes more reminiscent of the social sciences with its deep ideological divides than it is of the neurosciences.” We describe a data-driven theoretical framework for lightness/brightness research that resolves many of the definitional and theoretical confusions that have plagued communication between the various groups and impeded progress in the field. The proposed framework is based on two testable explanatory concepts. The first is the idea that much of the debate and confusion in the brightness/lightness literature stems from the fact that the term lightness, defined as apparent reflectance, is underspecified with regard to illumination and thus, due to the inverse problem, is often inadvertently used to refer to three very different and independent types of judgments that are not comparable (Blakeslee et al., 2008; Blakeslee and McCourt, 2012). Experimental support for this idea is provided by the data of Arend and Spehar (1993a,b); and Blakeslee et al. (2008). These studies demonstrate that under stimulus conditions where there is a visible illumination component (e.g., a shadow, transparency, or spotlight) observers can distinguish and match three independent dimensions of achromatic experience: apparent intensity (brightness), apparent local intensity ratio (brightness-contrast), and apparent reflectance (lightness). In the absence of a visible illumination component, however, achromatic visual experience reduces to two dimensions and, depending on stimulus conditions and observer instructions, judgments of apparent reflectance (lightness) are identical to judgments of apparent intensity (brightness) or apparent local intensity ratio (brightness-contrast). The second novel but closely related concept is that because apparent reflectance (lightness) judgments are based on different task-dependent information under different stimulus and/or instruction conditions, they can differ greatly in their degree of difficulty (Blakeslee and McCourt, 2012). This idea has the potential to explain the large variability in the accuracy of lightness judgments (i.e., the degree of “lightness constancy”) observed across studies (Gilchrist, 2006; Kingdom, 2011, 2013).

## Definitional confusions

As a first step toward the goal of building a common framework within which to advance the lightness/brightness literature, it is critical to understand the differences and confusions in the field that are related to terminology and to establish a common vocabulary (Blakeslee et al., 2008; Blakeslee and McCourt, 2012; McCann et al., 2014). These terminological differences are serious because they obscure both the fundamental nature of the inverse problem as well as the mechanisms the visual system employs to solve it. While we limit our discussion to the terms (and their definitions) that are most commonly encountered in the current literature to describe the psychological correlates of the three physical stimulus dimensions of intensity, reflectance, and illumination, other terms and definitions are also encountered sporadically in the literature, and care must be taken to translate the meanings of these terms into a common language.

The Commission Internationale de L'Eclairage (CIE, 1970) defines the term *brightness* as the attribute of a visual sensation according to which a given visual stimulus appears to be more or less intense, or according to which the area in which a visual stimulus is presented appears to emit more or less light (Wyszecki and Stiles, 1982; Wyszecki, 1986). *Lightness*, on the other hand, is defined (CIE, 1970) as the attribute of a visual sensation according to which the area occupied by the visual stimulus appears to emit more or less light *in proportion to that emitted* by a similarly illuminated area perceived as a “white” stimulus (Wyszecki and Stiles, 1982; Wyszecki, 1986). In describing the appearance of light (here restricted to a discussion of achromatic colors), the distinction between unrelated and related colors is also informative. According to Pokorny et al. (1991) unrelated colors refer to stimuli presented alone in a dark field or alternatively that fill the entire visual field (ganzfeld). Unrelated colors vary only in apparent intensity (brightness). Related colors, on the other hand, are those that are presented with other colors and have both an apparent intensity (brightness) and an apparent intensity relative to a similarly illuminated area perceived as a “white” stimulus (lightness). In other words, the dimension of lightness emerges only *in relation* to other colors.

Although the CIE definitions of brightness and lightness are still used, it has become more common in the recent psychophysical literature to use the definitions first suggested by a small group of researchers known as the Trieste group (Arend, 1993), who defined brightness as apparent luminance and lightness as apparent reflectance. Although the CIE and Trieste group definitions of brightness appear very similar (luminance after all is a photometric measure of physical intensity), it is worth noting that this term has come to be used quite differently by various research groups. For example, research groups focused on the elucidation of “low-level” mechanisms tend to be interested in brightness (apparent intensity) in general, and in brightness illusions in particular, because these phenomena are thought to result from (and therefore reveal the nature of) early neural processes such as photoreceptor light adaptation, retinal and early cortical luminance and contrast gain control, and spatial and temporal filtering by retinal, LGN, and early cortical receptive fields. In other words, brightness refers to an appearance-based percept that is the outcome of significant early processing by the visual system. These researchers would certainly agree with the long held assertion that although brightness (apparent intensity) is highly correlated with the physical stimulus intensity (luminance) for stimuli presented alone in a dark field (i.e., unrelated stimuli), it is nonetheless highly dependent on context for stimuli presented in a display containing multiple stimuli (i.e., related stimuli), and may differ greatly from photometric luminance (Wyszecki and Stiles, 1982; Wyszecki, 1986; Pokorny et al., 1991; Kingdom, 2011). Groups seeking explanations based on “higher-level” processes, on the other hand, tend to be interested in lightness (apparent reflectance) and, to the extent that they are concerned with neural mechanisms at all, focus on processes that support contrast coding. Contrast-coding mechanisms are of interest to these lightness researchers because they perform the types of computations that are thought to contribute to one form of “lightness constancy” (i.e., constancy over illumination), which refers to the ability of an observer to recognize that two surfaces under different illuminations have the same reflectance. Note that these contrast-coding mechanisms clearly overlap with the early neural mechanisms thought to underlie brightness. Considering that intensity (luminance) and reflectance are perfectly correlated for surfaces under homogeneous illumination, the fact that brightness (apparent intensity) and lightness (apparent reflectance) matching are also highly correlated under these conditions (Arend and Spehar, 1993a,b; Blakeslee et al., 2008; Rudd, 2010; Blakeslee and McCourt, 2012) is not surprising, and suggests a common underlying mechanism. While some researchers are comfortable acknowledging this theoretical overlap (Kingdom, 2011, 2013), other groups (Gilchrist et al., 1999; Gilchrist, 2006, 2007) are not, and despite accepting the similar definition of brightness as apparent luminance, have nonetheless (mis)interpreted the term “brightness” to mean “*the perception of a proximal quality—the raw intensity of some part of the image*” (Gilchrist, 2006, p. 6). Based on this misunderstanding they argue that “*the human visual system evolved to determine object properties, like lightness, illumination level, and 3D form, which are adaptive for survival, not proximal qualities like brightness, which are not*” (Gilchrist, 2006, p. 338). These quotations clearly reveal that this research group has equated the term brightness with the raw (unprocessed) intensity distribution incident on the retina. However, since observers lack access to the unprocessed intensity distribution (photoreceptor light adaptation, neural luminance and contrast gain control, and spatial and temporal filtering intervene at the earliest levels of the visual system) this is clearly a misunderstanding or misrepresentation of the term brightness (apparent intensity) as commonly (and historically) used by other researchers. As elaborated below, this confusion stems in part from a failure to appreciate the distinction between situations where apparent reflectance (lightness) judgments are based strictly on appearance (and are therefore identical to brightness or brightness-contrast judgments), vs. when they are based on an inferential judgment of lightness (i.e., when they are not based *directly* on appearance, and are independent of brightness and brightness-contrast).

Confusion regarding the meaning of the term lightness, however, is more serious and widespread. The CIE (1970) definition of lightness (perceived intensity relative to a *similarly illuminated* stimulus perceived as white) is highly correlated with the physical reflectance of a surface under the specified stimulus condition of similar illumination. Recall that because of the inverse problem reflectance can only be determined or estimated (correctly or incorrectly) based on either direct knowledge or prior assumptions (conscious or unconscious) about the illumination of the target surface or object. In other words, by specifying similar illumination the confounding influence of reflectance and illumination in determining the retinal intensity distribution (the inverse problem) is resolved. Note, however, that the specification of “similarly illuminated” also means that the CIE definition of lightness fails to pertain to other stimulus conditions such as comparisons between shadowed and un-shadowed regions. The definition of lightness as apparent reflectance (Arend, 1993), however, is also problematic since it ignores the inverse problem completely by referring directly to a physical property of the stimulus, its reflectance, without further qualification regarding the illumination. As mentioned previously, the data of Arend and Spehar (1993a,b) and Blakeslee et al. (2008), demonstrate that lightness, defined simply as apparent reflectance, is under-specified and may refer to three very different types of judgments. Under stimulus conditions where there is a visible illumination component (e.g., a shadow, transparency, or spotlight) observers can distinguish and match three independent dimensions of achromatic experience: apparent intensity (brightness), apparent local intensity ratio (brightness-contrast), and apparent reflectance (lightness). In the absence of a visible illumination edge, however, achromatic visual experience is reduced to two dimensions and, depending on stimulus conditions and observer instructions, judgments of apparent reflectance (lightness) are identical to judgments of apparent intensity (brightness) or apparent local intensity ratio (brightness-contrast) (see also Rudd (2010) for partial confirmation of these results). The terms inferred-lightness (Blakeslee and McCourt, 2003; Blakeslee et al., 2008) and projective-lightness (Reeves et al., 2008) have been introduced to distinguish lightness judgments where the illumination component in a scene (such as a shadow or spot-lit region) has been taken into account, and the lightness judgment is therefore not directly based on the appearance of the region or object to be judged, but is instead an estimate of how that region or object would appear when not in shadow or spot-light. Note that this type of lightness judgment requires that the observer first estimate the illumination component in the scene. This is possible, however, only when the illumination component is clearly visible allowing the observer to compare the brightness information (brightness-contrast) on either side of the illumination boundary (as it crosses objects or surfaces in the scene) to determine its magnitude. As discussed in more detail below, Blakeslee and McCourt (2012) suggest that inferred-lightness judgments can vary greatly in their difficulty ranging from easy (seemingly automatic) to difficult (requiring focused effort).

## How confusion of the three types of lightness judgments has distorted the literature: examples

It is critically important to understand that judgments of inferred-lightness, made possible (and optimal) by the existence of a visible illumination component, are entirely different from the appearance-based (and optimal) judgments of apparent reflectance (lightness) that are identical to judgments of apparent intensity (brightness) under conditions of homogeneous illumination, and to apparent local intensity ratio (brightness-contrast) under conditions where illumination varies over time (or space) but no information from a visible illumination boundary is available. Blakeslee et al. (2008) and Blakeslee and McCourt (2012) argued that a failure to make this vital distinction is responsible for a great deal of the current confusion and division in the field. The three types of lightness judgments are in fact *not* comparable but are frequently unwittingly conflated due to the underspecified definition of lightness as apparent reflectance. This confusion is compounded by the inconsistent recognition by observers, and often experimenters as well, of the type of information on which their lightness judgments are based, and of when an independent dimension of inferred-lightness is actually available for matching. This is especially important to be aware of when comparing the results of studies involving Munsell paper displays and matching arrays with studies using electronic stimuli and matching procedures. For example, Munsell matching to an array of papers ranging from black to white is common practice in lightness research and, like luminance matching in brightness research, may index any one of the three types of lightness judgments depending on the matching conditions. If the matching chips and stimulus display are homogeneously illuminated at the same level, then optimal lightness matching would be equivalent to brightness (i.e., apparent intensity) matching. More common in paper matching studies, however, has been the practice of using a different illumination level on the matching chips. This procedure makes some form of brightness-contrast or comparison matching (not necessarily between adjacent regions) optimal for lightness matching. In the presence of a visible illumination, however, the optimal lightness match to a location within the shadow or spotlight is an inferential-lightness match.

Blakeslee et al. (2008) and Blakeslee and McCourt (2012) investigated this issue with regard to a history of data on simultaneous contrast, in which discrepancies in the magnitude and direction of the reported lightness effects appeared, in some instances, to be directly the result of comparing disparate types of lightness judgments, i.e., those based on appearance (and the same as brightness or brightness-contrast) with those based on inference (Gilchrist, 1979, 1988; Gilchrist et al., 1983) and, in other instances, to observers erroneously judging brightness or brightness-contrast rather than the available and independent dimension of inferred- (projective-) lightness (Gilchrist et al., 1999; Gilchrist, 2006). For example, Gilchrist (Gilchrist, 1979; Gilchrist et al., 1983) produced several variants of the classic simultaneous contrast stimulus by using illumination rather than reflectance differences to produce the different backgrounds. Gilchrist and colleagues reported that a mid-gray background was made to appear white on one side and black on the other by illuminating half of the background such that it was 30 times more luminous than the shadowed half (thus mimicking the 30:1 intensity ratio of white vs. black paper under homogeneous illumination). Likewise, in order to equate the luminances of the target stimuli, as in the classic simultaneous contrast display, the target on the illuminated side was made of black paper, such that it reflected 30 times less light than a white paper target on the shadowed side. In this way, these studies claimed to reproduce the retinal light intensities that would result from viewing a reflectance-based simultaneous contrast stimulus (i.e., equiluminant mid-gray targets on black and white backgrounds) under homogeneous illumination. When this illumination-edge stimulus was viewed through a rectangular aperture that masked everything but the targets and their near backgrounds (Gilchrist, 1979), or in a manner that obscured the actual illumination conditions (Gilchrist et al., 1983), the display was reported to look like a standard simultaneous contrast display. Under these conditions the authors state that observers described the illumination edge as a reflectance edge between black and white backgrounds and matched the lightness of the targets to mid-gray (the one on the bright background slightly darker than the one on the dark background due to brightness induction). However, when observers viewed the display without the aperture (Gilchrist, 1979), or when an additional background was inserted which revealed the illumination so that observers could clearly see and describe the differential illumination on the two halves of the stimulus (Gilchrist et al., 1983), they matched the lightness of the illuminated target to black and the lightness of the shadowed target to white. Thus, the lightness (apparent reflectance) of the two targets was reported to be profoundly different in this condition even though the intensity of light coming from each target and near background remained the same. Gilchrist (Gilchrist, 1979; Gilchrist et al., 1983) interpreted this result as evidence that, when given enough information, the visual system can classify edges into those due to illumination vs. those due to reflectance prior to integrating the reflectance edges to determine the reflectance of various regions.

Based on these reports and on several subsequent studies (Arend and Goldstein, 1987; Schirillo et al., 1990; Arend and Spehar, 1993a,b; Blakeslee et al., 2008) investigated the possibility that two different types of lightness judgments were being erroneously compared in the Gilchrist experiments. Blakeslee et al. (2008) measured apparent intensity (brightness), brightness-contrast, and apparent reflectance (lightness) in simultaneous contrast stimuli that were produced by rendering in a virtual reality environment, and by using calibrated neutral value Munsell papers (matte) and projected light. The results from these studies indicated that when illumination appeared homogeneous, or when observers were instructed that illumination was homogeneous (for example, see Figure 1A), observer brightness matches were identical to their lightness matches. Under conditions where an illumination edge was clearly visible, however, (for example, see Figure 1B) although observer brightness matches remained similar to those in the homogeneous illumination condition, their lightness matches differed significantly from their brightness matches. In other words, under these conditions subjects were able to make independent inferred-lightness judgments. On the basis of these findings Blakeslee et al. (2008) concluded that the profound lightness effects reported to be produced by the type of background edge in the earlier Gilchrist experiments (Gilchrist, 1979, 1988; Gilchrist et al., 1983) were likely due to two different stimulus dimensions being matched in the two conditions. That is, subjects matched lightness using apparent intensity (brightness) or brightness-contrast (depending on the illumination conditions for the matching chips) in the reflectance edge condition, but used the independent dimension of inferred-lightness in the condition where an illumination edge was clearly visible. In other words, it is clear from the results of Blakeslee et al. (2008) that although the inferred-lightnesses of the test patches in the visible-illumination condition were matched to black and white in the original Gilchrist experiments, they would not, as is usually assumed [for example see Kingdom (2003), p. 25; and Kingdom (2011), p. 662], have actually *appeared* black and white. Thus, what was reported as a profound lightness effect, and is assumed to be directly based on appearance by many investigators, is actually a confusion resulting from comparing a direct appearance-based judgment of lightness with an indirect inferential lightness judgment.

Interestingly, Gilchrist reported quite different results in later experiments designed to test the intrinsic image model described above against his newer anchoring model (Gilchrist et al., 1999; Gilchrist, 2006). In these experiments, which we refer to as “unequal-increment” experiments, a dark-gray target square was centered on one side, and a white target square was centered on the other side, of a black rectangular background. Following the application (to the half of the background containing the dark-gray target) of a clearly visible illumination component (spotlight) of sufficient intensity to make the dark-gray target within the spotlight possess the highest luminance, the dark-gray target was reported to be judged as significantly lighter than the white target outside the spotlight. Gilchrist (Gilchrist et al., 1999; Gilchrist, 2006) pointed out that this result was exactly opposite to that predicted by the intrinsic image explanation and instead supported his anchoring model of lightness perception. Note that a model that accounted for the illumination component in the scene (like the intrinsic image explanation) would predict that the lightness (apparent reflectance) of the illuminated target would be similar to the target's actual reflectance (i.e., dark-gray), and likewise for the lightness of the target outside the spotlight (i.e., white) (Gilchrist et al., 1999; Gilchrist, 2006). The anchoring model, on the other hand, predicts that the lightness of the dark-gray target in the spotlight will be matched to white because it is the highest luminance in its local framework, as well as in the global framework. The lightness of the white target outside the spotlight is predicted to be matched to light middle-gray because, although it is the highest luminance in its local framework, it is not the highest luminance in the global framework (Gilchrist et al., 1999; Gilchrist, 2006). Blakeslee et al. (2008) measured brightness and lightness judgments in this unequal-increment stimulus and concluded, based on their results, that the observers in this later Gilchrist experiment, when asked to judge lightness (Gilchrist et al., 1999; Gilchrist, 2006), were instead “erroneously” basing their judgments on appearance (i.e., brightness or brightness-contrast), rather than on the available independent dimension of inferred-lightness. Had inferred-lightness been judged in this experiment, as it was in the earlier edge-substitution studies, the opposite conclusion, i.e., support for the intrinsic image model as opposed to the anchoring model, would have been reached!

**Figure 2:**
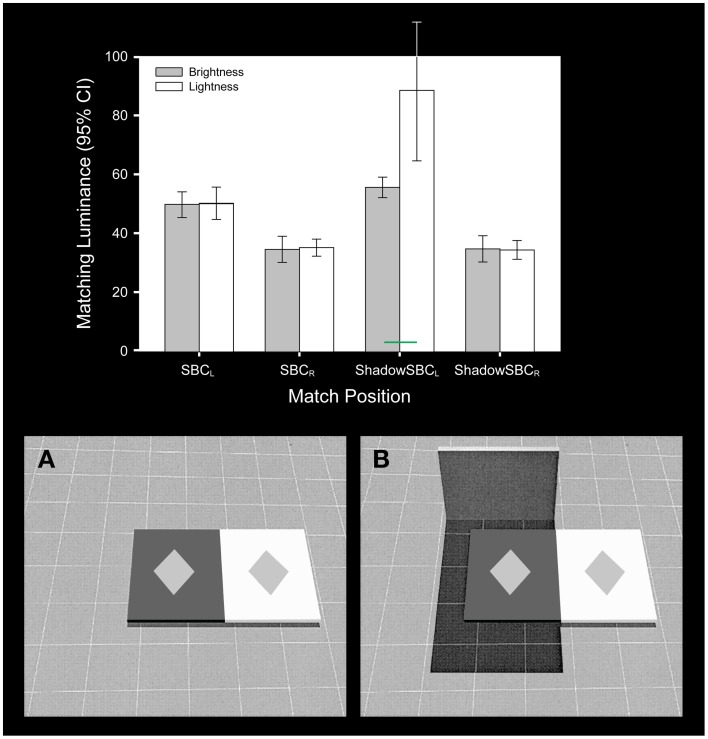
**The simultaneous contrast stimulus used by Blakeslee and McCourt (2012)**. The stimulus was modeled on the Williams et al. (1998) version of a reflectance edge **(A)** and an illumination edge **(B)** simultaneous contrast illusion. In **(B)** the test patches and near backgrounds are identical to those in **(A)**; however, a dark far surround has been added that causes the left half of the stimulus to appear to be in shadow. The bar graph plots the mean (and 95% confidence intervals) of four observers brightness (gray bars) and lightness (white bars) matches for each test patch within the stimulus displays. The test patches are labeled from left to right in the order that they appear in the stimuli. Lightness matches only differed significantly from brightness matches (green bar) in **(B)** where the test patch was seen beneath a shadow. Under these conditions subjects were able to make independent inferred judgments of lightness.

**Figure 3:**
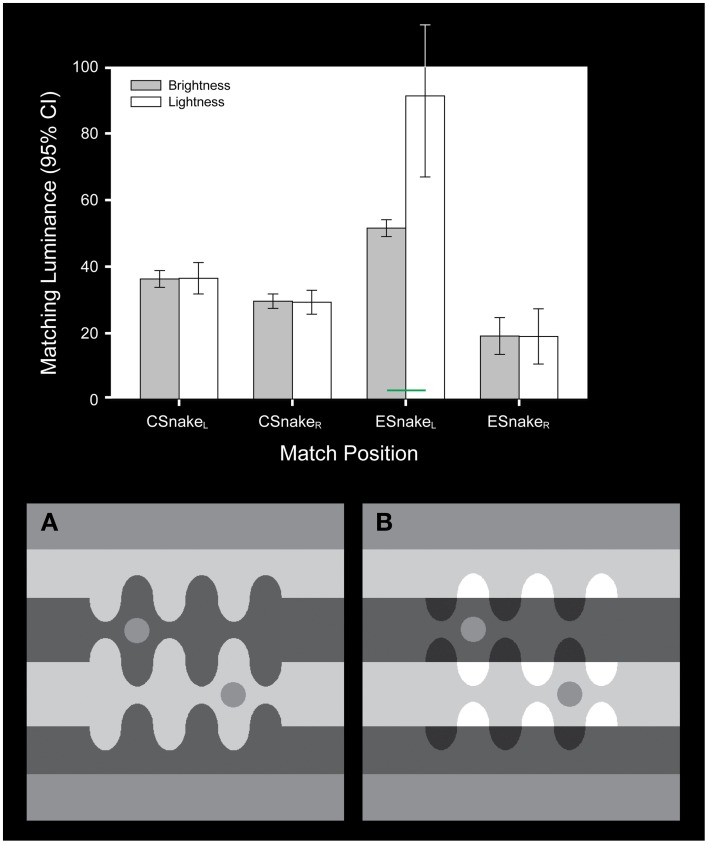
**The snake illusion stimulus used by Blakeslee and McCourt (2012)**. The stimulus is modeled on Adelson (2000). The test patches in **(A, B)** share the same luminance. In addition, the upper test patches in both stimuli have the same lower background luminance and the lower test patches share the same higher background luminance. The stimuli differ in the luminances of the more distant regions (the snake undulations) such that the upper test patch in **(B)** appears to lie beneath a transparent overlay. The bar graph plots the mean (and 95% confidence intervals) of four observers brightness (gray bars) and lightness (white bars) matches for each test patch within the stimulus displays. The test patches are labeled left to right in the order that they appear in the stimuli. Lightness judgments differed significantly from brightness judgments (green bar) only for the upper test patch in **(B)** where the test patch was seen beneath a transparency. Under this condition subjects were able to make independent inferred judgments of lightness.

**Figure 4:**
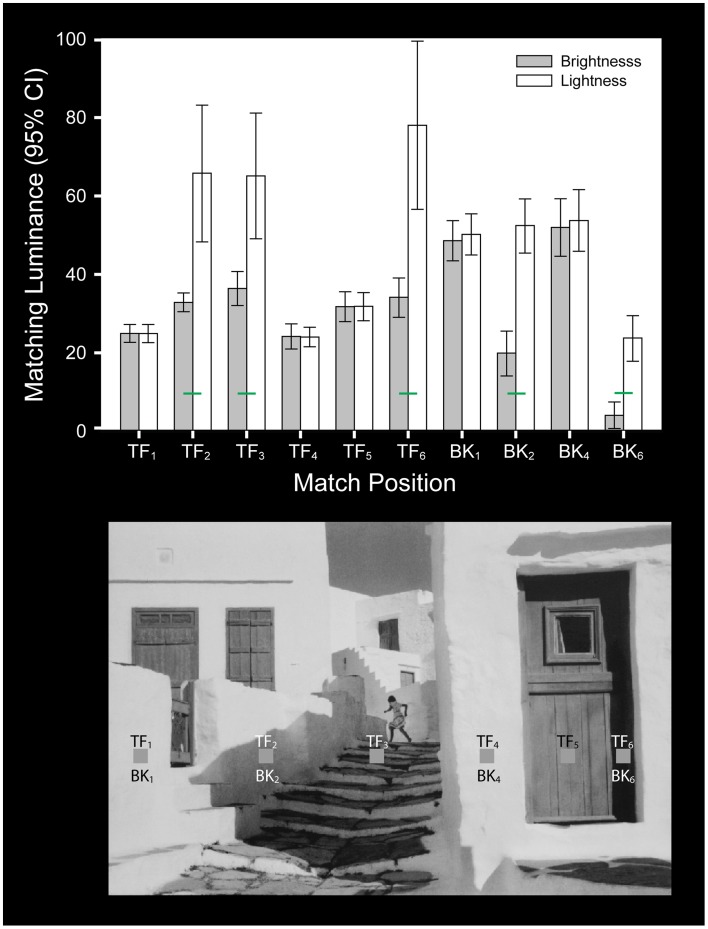
**Cartier-Bresson photograph**. An image of the Cartier-Bresson stimulus used by Blakeslee and McCourt (2012). The test patches are labeled from left to right (TF_1_–TF_6_). In addition, four background regions located below the test patches were selected for matching and are labeled: BK_1_, BK_2_, BK_4_, and BK_6_. The luminance of all of the test patches is identical, however, their backgrounds differ. The bar graph plots the mean (and 95% confidence intervals) of four observers brightness (gray bars) and lightness (white bars) matches for each test patch and background patch within the stimulus. Lightness matches differed significantly from brightness matches (green bars) only at locations where the test patch was seen beneath a shadow. Under these conditions subjects were able to make independent inferred-lightness judgments.

Blakeslee and McCourt (2012) extended the investigation of brightness (apparent intensity) and lightness (apparent reflectance) matching to a number of other well-known visual stimuli that contained a visible illumination component (i.e., a shadow or transparent overlay) as well as to their homogeneously illuminated control stimuli. These stimuli included: (1) the Williams et al. (1998) version of the illumination edge verses reflectance edge simultaneous contrast illusion (Figure 2) (Gilchrist et al., 1983; Williams et al., 1998; Purves et al., 2004); (2) the snake illusion (Figure 3) (Somers and Adelson, 1997; Adelson, 2000); (3) a paint/transparency/shadow checkerboard illusion derived from Adelson's checkershadow illusion (Adelson, 1995); (4) the paint/shadow illusion (Hillis and Brainard, 2007); (5) the argyle illusion (Adelson, 1993); (6) the wall of blocks illusion (Adelson, 1993; Logvinenko, 1999); and (7) a Cartier-Bresson photograph of a natural scene containing shadowed regions (Figure 4) (similar to the Cartier-Bresson photograph used by Gilchrist (2006) and Gilchrist and Radonjic (2010). These stimuli were chosen, in part, because similar to the investigations of simultaneous contrast discussed above, the previous investigations of these effects also appeared to be confusing brightness (apparent intensity) and lightness (apparent reflectance) under the various conditions.

The significant main effects and interactions associated with the apparent intensity (brightness) and apparent reflectance (lightness) matching behavior in the Blakeslee and McCourt (2012) study confirmed for this important set of stimuli that while the two types of judgments were not significantly different under homogeneous illumination, they differed significantly when one of the test patches was seen beneath a transparent overlay or in shadow (noted by green statistical comparison bars in Figures 2–4). Under these conditions subjects with clear instructions regarding the difference between brightness and lightness matching were able to make inferential (Blakeslee and McCourt, 2003; Blakeslee et al., 2008; Reeves et al., 2008) judgments of lightness by using the information provided by the brightness-contrast at the visible illumination edge to account for the shadow or transparency and estimate the reflectance of the underlying surface. As mentioned previously, Blakeslee and McCourt (2012) made the additional argument that under some circumstances inferred-lightness judgments appear to be effortless and accurate, for example where a shadow or transparent overlay falls across only a portion of an object or surface, allowing its apparent reflectance (lightness) to be judged based on those parts of the object or surface lying outside of the shadowed or transparency occluded region. This strategy for judging inferred-lightness is probably a highly overlearned behavior and is illustrated in Figure 4 for the area labeled BK_2_, where observers were asked to match the apparent reflectance (lightness) of a shadowed part of the wall, but where a neighboring part of the same wall not in shadow is also clearly visible. Note that the variability of the mean lightness matches across observers for this region (BK_2_) is small and comparable to that for brightness matching. Under other conditions, where an object or surface is completely shadowed or under a transparent overlay, such that its illumination must be estimated based on a comparison of brightness-contrast at a remote illumination edge, however, inferential lightness judgments are considerably more effortful and imprecise. This is illustrated in Figure 4 where observers were asked to match the apparent reflectance (lightness) of the shadowed equiluminant test fields labeled TF_2_, TF_3_, and TF_6_. The variability of lightness matches across observers in this condition is much greater than for BK_2_. Although not illustrated in the figures, the within-subjects variability of the inferred-lightness matches mirrored these between subject results.

The confusion here is that with the exception of the edge-substitution experiments discussed earlier (Gilchrist, 1979, 1988; Gilchrist et al., 1983), previous studies of the illusions investigated by Blakeslee and McCourt (2012) were all interested in appearance effects, i.e., the apparent intensity (brightness) of the target regions and deliberately instructed subjects to match brightness (apparent intensity). These same studies, however, discussed and interpreted these illusions using the term lightness (Adelson, 2000; Logvinenko and Ross, 2005; Gilchrist, 2006; Todorovic, 2006; Hillis and Brainard, 2007; Kingdom, 2011). Clearly this use of terms is justifiable (albeit potentially confusing) under conditions of homogeneous illumination where apparent intensity (brightness) and apparent reflectance (lightness) matching yield the same result. However, referring to appearance-based brightness judgments as lightness judgments in the stimuli actually containing a visible illumination component is a seriously confusing misnomer since independent inferred-lightness judgments are in fact possible in these stimuli. This confusing naming error may have persisted in part because it has only rarely been the case that both lightness and brightness have been measured under the same conditions in the same experiment (Schirillo et al., 1990; Arend and Spehar, 1993a,b; Blakeslee et al., 2008; Rudd, 2010; Blakeslee and McCourt, 2012), and also because as yet there is not a general appreciation of when the terms brightness (or brightness-contrast) and lightness refer to the same or different dimensions of achromatic experience.

Paradoxically, however, this difference between the dimension of inferred-lightness and the dimension of brightness is well accepted for relatively easy inferred-lightness judgments, for example as discussed above, when a shadow is cast across a surface but where a neighboring part of the same surface not in shadow is also visible (see Figure 4, area labeled BK_2_). Indeed, a stimulus of this type is the textbook illustration of the difference between lightness and brightness (for example see Palmer, 1999; Kingdom, 2011). It is ironic, therefore, that this is also a stimulus that the anchoring model of lightness is challenged to explain (Gilchrist et al., 1999; Gilchrist, 2006; Zdravkovic et al., 2006). Gilchrist (2006) refers to this particular challenge as the “response paradox” and writes, “*when an obvious cast illuminance edge crosses a region of homogeneous reflectance, an intractable ambiguity is produced in the data, if not the percept itself. Observers report that the lightness is the same on both sides of the illuminance edge, but when asked to make matches from a Munsell chart, all of the same observers assign different numbers to the two sides*. *This cannot be dismissed as confusion between lightness and brightness: expert observers show the same paradox.”* The problem for the anchoring model is that local anchoring within illumination frames predicts two values for the lightness of the object, one for the part of the object (patch) in the brighter illumination and one for the part of the object (patch) in the dimmer illumination. Zdravkovic et al. (2006) conducted a number of experiments to study the relationship between patch matches and whole object matches. They concluded that the object match represented a compromise between the match for the patch in the field of highest illumination and the patch in the largest field of illumination, and proposed that these new rules be applied as an extension to the anchoring model but only for the case of multi-lit patches. A close examination of these results, however, makes it clear that the “response paradox” is easily and parsimoniously resolved by using the framework we propose. In other words, what are being called the “lightness” matches to the parts of the objects (patches) under the two different levels of illumination are direct appearance-based matches (i.e., brightness or brightness-contrast matches depending on the matching conditions). The “lightness” matches made for the whole object, however, are based on inferred-lightness. In other words, the object in this instance, by definition, has a single reflectance value but two regions of very different brightness (apparent intensity) due to the unequal levels of illumination. The new rules proposed to rescue the anchoring model in the case of multi-lit patches, in essence, describe which region is seen as shadowed or spot-lit as opposed to “normally-lit” and thereby inform something akin to an inferred-lightness judgment for the whole object.

**Figure 5:**
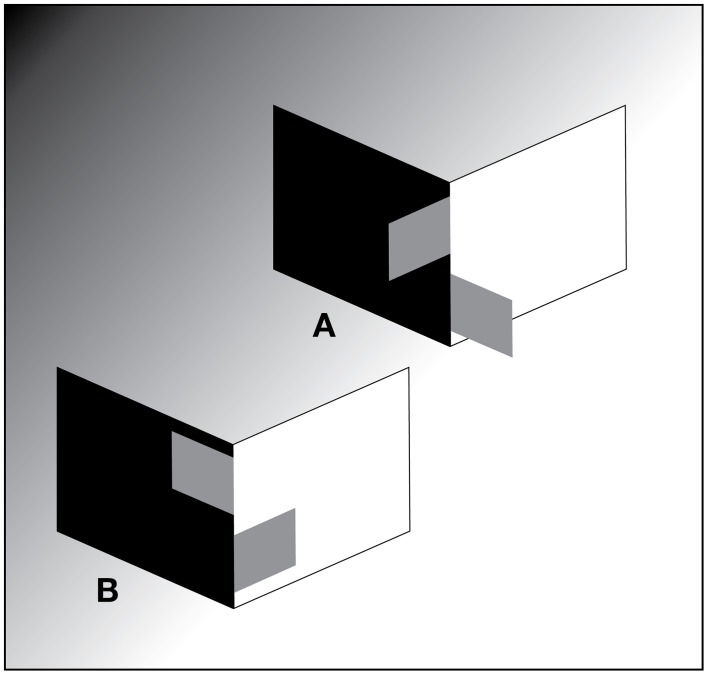
**Illustration depicting the stimulus configuration employed by Radonjic et al. (2010) and Radonjic and Gilchrist (2013) to reexamine the effect of depth on lightness matching**. The upper target (covered with black paper) extended from the white side of the cube such that it was seen against the black side. The lower target (covered with white paper) extended from the black side of the cube such that it was seen against the white side. The display was lit from the right such that the white side of the cube and the black target extending from it were highly illuminated, while the black side of the cube and the white target extending from it were shadowed and dimly illuminated. Illumination was adjusted so that the upper (black) target and the lower (white) target were equiluminant. When viewed binocularly **(A)** the targets were seen veridically in depth and therefore also in illumination. When viewed monocularly, however, [as depicted in **(B)**] the targets appeared to switch depth planes (due to a loss of depth information) and therefore also switched their perceived illumination.

Similar confusion resulting from a lack of distinction between lightness judgments based directly on appearance (i.e., that are the same as brightness or brightness-contrast), and those based on inferred-lightness, is also contaminating recent work re-examining the effect of depth on lightness (Radonjic et al., 2010; Radonjic and Gilchrist, 2013). The stimuli employed in these studies are variants of the perpendicular planes stimuli used in earlier studies by Gilchrist (1977, 1980) and purport to extend his previous work showing a large effect of perceived illumination (manipulated by the perceived depth of the target) on lightness judgments under conditions in which the surround of the target remains the same. Although the stimuli are different, the logic of these experiments is similar to the reflectance vs. illumination edge simultaneous contrast experiments discussed earlier. Here, however, perceived depth is manipulated to make the target appear to be in one of two different spatial positions and therefore part of an illuminated (spot-lit) or non-illuminated (shadowed) region of the display (Figure 5). Briefly, observers viewed what appeared to be a cube oriented such that a vertical right angle pointed toward the observer. The left side of the cube was covered with black paper and the right side of the cube was covered with white paper. Extending from the corner was an upper and a lower trapezoidal shaped target. The upper target (covered with black paper) extended from the white side of the cube such that it was seen against the black side of the cube. The lower target (covered with white paper) extended from the black side of the cube such that it was seen against the white side of the cube. In addition, the display was lit from the right such that the white side of the cube and the black target extending from it were highly illuminated, while the black side of the display and the white target extending from it were shadowed and therefore dimly illuminated. Importantly, this illumination was adjusted so that the upper (black) target and the lower (white) target were actually equiluminant. When viewed binocularly (as depicted in Figure 5A) the targets were seen veridically in depth and therefore also in illumination. When viewed monocularly, however, (as depicted in Figure 5B) the targets appeared to switch planes (due to a loss of depth information) and therefore also switched their apparent illumination. In the binocular (veridical illumination) condition the mean Munsell match to the upper (black and illuminated) target was close to black (mean Munsell value = 2.85; black = Munsell 2.0) and the lower (white and non-illuminated) target was matched to light gray (mean Munsell value = 7.6). In the monocular condition, however, the upper (black and illuminated) target, now seen as lying on top of the black and non-illuminated side of the cube, was matched to middle gray (mean Munsell value = 5.2) and the lower (white and non-illuminated) target, now seen as lying on top of the illuminated white side of the display, was matched to dark gray (mean Munsell value = 3.6). We again suggest that the lightness matches in the binocular conditions were based on inferred-lightness while those in the monocular conditions were based directly on appearance (i.e., were the same as judgments of brightness or brightness-contrast) and would have remained the same across conditions. We can be reasonably confident of this interpretation because, despite the lack of brightness or brightness-contrast matching data, observers in both of these studies (Radonjic et al., 2010; Radonjic and Gilchrist, 2013) were informed as to the differences between lightness and brightness and instructed specifically to make lightness matches, “*to pick the chip from the chart that is the same actual color as the target; that is, cut from the same piece of paper as the target*.” In addition, observer matches were reported to be excluded from the analysis if: (1) the observer failed to perceive the intended spatial position of the targets when questioned as to the appearance of the display; (2) during debriefing it was established that the observer was making brightness and not lightness matches (e.g., “I saw the target as white, but it appeared darker; I matched how it appeared”); and (3) the matches fell more than three standard deviations above or below the mean of the whole group in a given condition.

## Summary

We have presented, and provided data to support, a framework for lightness and brightness research that resolves confusions and paradoxes in the literature, and provides insight into the mechanisms the visual system employs to tackle the inverse problem. The main idea is that much of the debate and confusion in the literature stems from the fact that lightness, defined as apparent reflectance, is underspecified with regard to illumination and thus, due to the inverse problem, is often used to refer to three very different and independent types of judgments that are not comparable. Experimental support for this idea is provided by the data of Arend and Spehar (1993a,b) and Blakeslee et al. (2008). These studies demonstrated that under stimulus conditions containing a visible illumination component, such as a shadow boundary, observers can distinguish and match three independent dimensions of achromatic experience: apparent intensity (brightness), apparent local intensity ratio (brightness-contrast), and apparent reflectance (lightness). In the absence of a visible illumination boundary, however, achromatic vision reduces to two dimensions and, depending on stimulus conditions and observer instructions, judgments of lightness are identical to judgments of brightness or brightness-contrast. Due to the inverse problem, employing the optimal strategy to judge reflectance depends on the observer having some knowledge of (or assumptions about) the illumination. While it is clear that observers are capable of making optimal judgments of lightness when specifically instructed to do so under laboratory conditions (Arend and Spehar, 1993a,b; Blakeslee et al., 2008; Blakeslee and McCourt, 2012), it is unclear to what extent these optimal strategies are employed in other laboratories or in natural vision. Furthermore, because optimal lightness judgments are based on different information under the various illumination conditions, they can vary in their degree of difficulty and in their accuracy. Consideration of these factors may, in part, explain the large variability in the reported accuracy of lightness judgments (i.e., the degree of lightness constancy) observed across studies.

### Conflict of interest statement

The authors declare that the research was conducted in the absence of any commercial or financial relationships that could be construed as a potential conflict of interest.
